# Sulforaphane (SFA) protects neuronal cells from oxygen & glucose deprivation (OGD)

**DOI:** 10.1371/journal.pone.0248777

**Published:** 2021-03-18

**Authors:** Zeenat Ladak, Elizabeth Garcia, Jenny Yoon, Takaaki Landry, Edward A. Armstrong, Jerome Y. Yager, Sujata Persad

**Affiliations:** Faculty of Medicine & Dentistry, Department of Pediatrics, University of Alberta, Edmonton, Alberta, Canada; University of Louisville, UNITED STATES

## Abstract

**Background:**

Perinatal brain injury results in neurodevelopmental disabilities (neuroDDs) that include cerebral palsy, autism, attention deficit disorder, epilepsy, learning disabilities and others. Commonly, injury occurs when placental circulation, that is responsible for transporting nutrients and oxygen to the fetus, is compromised. Placental insufficiency (PI) is a reduced supply of blood and oxygen to the fetus and results in a hypoxic-ischemic (HI) environment. A significant HI state in-utero leads to perinatal compromise, characterized by fetal growth restriction and brain injury. Given that over 80% of perinatal brain injuries that result in neuroDDs occur during gestation, prior to birth, preventive approaches are needed to reduce or eliminate the potential for injury and subsequent neuroDDs. Sulforaphane (SFA) derived from cruciferous vegetables such as broccoli sprouts (BrSps) is a phase-II enzyme inducer that acts via cytoplasmic Nrf2 to enhance the production of anti-oxidants in the brain through the glutathione pathway. We have previously shown a profound *in vivo* neuro-protective effect of BrSps/SFA as a dietary supplement in pregnant rat models of both PI and fetal inflammation. Strong evidence also points to a role for SFA as treatment for various cancers. Paradoxically, then SFA has the ability to enhance cell survival, and with conditions of cancer, enhance cell death. Given our findings of the benefit of SFA/Broccoli Sprouts as a dietary supplement during pregnancy, with improvement to the fetus, it is important to determine the beneficial and toxic dosing range of SFA. We therefore explored, *in vitro*, the dosing range of SFA for neuronal and glial protection and toxicity in normal and oxygen/glucose deprived (OGD) cell cultures.

**Methods:**

OGD simulates, *in vitro*, the condition experienced by the fetal brain due to PI. We developed a cell culture model of primary cortical neuronal, astrocyte and combined brain cell co-cultures from newborn rodent brains. The cultures were exposed to an OGD environment for various durations of time to determine the LD50 (duration of OGD required for 50% cell death). Using the LD50 as the time point, we evaluated the efficacy of varying doses of SFA for neuroprotective and neurotoxicity effects. Control cultures were exposed to normal media without OGD, and cytotoxicity of varying doses of SFA was also evaluated. Immunofluorescence (IF) and Western blot analysis of cell specific markers were used for culture characterization, and quantification of LD50. Efficacy and toxicity effect of SFA was assessed by IF/high content microscopy and by AlamarBlue viability assay, respectively.

**Results:**

We determined the LD50 to be 2 hours for neurons, 8 hours for astrocytes, and 10 hours for co-cultures. The protective effect of SFA was noticeable at 2.5 μM and 5 μM for neurons, although it was not significant. There was a significant protective effect of SFA at 2.5 μM (*p<*0.05) for astrocytes and co-cultures. Significant toxicity ranges were also confirmed in OGD cultures as ≥ 100 μM (*p<*0.05) for astrocytes, ≥ 50 μM (*p<*0.01) for co-cultures, but not toxic in neurons; and toxic in control cultures as ≥ 100 μM (*p<*0.01) for neurons, and ≥ 50 μM (*p<*0.01) for astrocytes and co-cultures. One Way ANOVA and Dunnett’s Multiple Comparison Test were used for statistical analysis.

**Conclusions:**

Our results indicate that cell death shows a trend to reduction in neuronal and astrocyte cultures, and is significantly reduced in co-cultures treated with low doses of SFA exposed to OGD. Doses of SFA that were 10 times higher were toxic, not only under conditions of OGD, but in normal control cultures as well. The findings suggest that: 1. SFA shows promise as a preventative agent for fetal ischemic brain injury, and 2. Because the fetus is a rapidly growing organism with profound cell multiplication, dosing parameters must be established to insure safety within efficacious ranges. This study will influence the development of innovative therapies for the prevention of childhood neuroDD.

## Introduction

Perinatal brain damage refers to injury that occurs from approximately 24 weeks gestation to 1 month after birth. 80–90% of injuries that result in cerebral palsy and other developmental disabilities, occur in utero, before birth [1]. Current therapies focus on rescue rather than prevention. Currently, hypothermia is the only rescue therapy accepted as a standard of care, and targets only 10–20% of newborn injuries at term. It therefore does not address the majority of perinatal brain injuries (PBIs) [1,2]. The extent of injury depends on the underlying cause and gestational age at the time of injury [3]. There are multiple factors which contribute to PBIs such as placental insufficiency, inflammation, infection, toxicity, and genetics. This study focuses on PBI caused by placental insufficiency (PI) [4]. The placenta is a vital organ in fetal development as it is responsible for transporting nutrients and oxygen to the fetus from the mother [4]. PI manifests as an inadequate transfer of oxygen and nutrients to the fetus by a reduced supply of blood; this results in a hypoxic-ischemic (HI) environment for the fetus [5,6]. The type of PBI is dependent on the gestational age of the fetus at the time of injury and duration of PI. While a cortical gray matter injury would mainly affect neuronal and astroglial function in an infant closer to term, a white matter injury would more likely affect the oligodendroglia in the preterm infant before 34 weeks gestation. Not surprisingly, there is often an overlap of both gray and white matter damage [7–9].

PBI results in a spectrum of neurodevelopmental disabilities (neuroDDs) that have lasting effects on a child’s life [3]. These disabilities occur due to an injury of the central nervous system and can include learning and behaviour disorders, epilepsy, autism spectrum disorder (ASD), attention deficit hyperactive disorder (ADHD), and most notably, cerebral palsy (CP) [10,11]. CP is a permanent, non-progressive, musculoskeletal disorder, and is a common outcome of PBI. Despite an increasing focus on perinatal health, the incidence rate of CP has not decreased; it occurs in 2.5–4.0/1000 live term births and this rate increases to 16-22/1000 for prematurely born infants [12]. It is common for more than one neuroDD to be present in a child, and in addition to musculoskeletal problems, CP is often accompanied by disruptions in sensation, cognition, communication, and behaviour, including ASD and ADHD [1]. Hypothermia is an effective therapeutic strategy for brain injuries that occur at the time of birth; however, it has not been shown to decrease the overall incidence of neuroDDs [3]. A preventative approach is needed whereby treating the fetus via the mother prevents the occurrence or extent of PBI, and ultimately reduces the incidence of neuroDDs.

Isothiocyanate, 4-methylsulfinylbutyl, is commonly known as Sulforaphane (SFA), and is derived from cruciferous vegetables, most prominently, broccoli sprouts [13–16]. It is naturally found as a R-SFA isomeric form, which is more active than the S-SFA isomer. Due to rapid interconversion between the two enantiomers in a biological setting, the racemic mixture (50% of each enantiomer) can be used experimentally, and is hereby noted as SFA [13–16]. SFA is derived from a hydrolysis reaction between glucoraphanin and myrosinase which are both found as part of the broccoli sprout plant. SFA can also be formed by a similar reaction between glucoraphanin and the myrosinase in gut microbiota [17]. SFA has been proposed as a natural health product (NHP) that serves as an anti-oxidant, anti-inflammatory, and anti-cancer agent [15]. It has also been shown to be effective as a therapeutic for neurodegenerative disorders such as Alzheimer’s, Amyotrophic Lateral Sclerosis, and Parkinson’s disease [14,18]. SFA is a phase II enzyme activator and works through an anti-oxidative mechanism via the Nrf2/ARE pathway. The anti-oxidant response element (ARE) is responsible for increasing anti-oxidative enzymes, such as glutathione reductase, to neutralize reactive intermediates such as reactive oxygen species (ROSs) [13]. Its profound effect is related to the central role of glutathione as an endogenous anti-oxidant [19]. Activation of ARE is dependent on nuclear factor E2-related factor (Nrf2) translocating to the nucleus [13]. Nrf2 is normally suppressed in the cytoplasm when bound to Kelch-like ECH-associated protein 1 (KEAP1) [13,16,18,20]. Acting as an alkylating agent and electrophile, SFA oxidizes KEAP1 in order to release Nrf2 which can then migrate to the nucleus and activate ARE [13,15].

SFA has also shown promise in protecting the developing fetal brain in rat models of acute brain injury [13]. SFA is able to cross the blood-brain and placental barrier and our previous work has shown the potential for it as a neuroprotective agent in a perinatal setting [21]. Broccoli sprouts fed to pregnant rodent dams during the last trimester of pregnancy with a bilateral uterine artery ligation, showed protection of the fetal brain [1]. However, since the protective concentration of broccoli sprouts (BrSps) compared to SFA is approximately 400 mg/kg to 1 mg/kg respectively, it would not be ideal for a pregnant woman to eat such a large amount of BrSps daily. There is also a risk of salmonella poisoning with BrSps, making them not recommended to have during pregnancy. Since the mechanistic dominant protective effect of BrSps comes from SFA, it makes sense to maximize the value of BrSps in the form of a feasible treatment with SFA [21]. SFA is a hormetic compound, whereby it exhibits a biphasic dose response [22]. At a high dose, SFA has been shown to be pro-apoptotic and is anti-cancerous, while at a low dose it is anti-apoptotic and protective [22]. It should be noted that like cancer, the fetus is also a rapidly dividing organism. Understanding the boundaries of therapeutic benefits and toxicity are therefore necessary to move this potential therapy forward to clinical use [23].

In this study, we explored the effects of SFA, and determined a safety and toxicity profile of SFA across the spectrum of brain cells. PI leads to an HI environment in utero which can result in the increase production of ROSs; here we used oxygen and glucose deprivation (OGD) *in vitro* to mimic the effects of PI [3]. We hypothesize that a low dose of SFA will be protective against OGD-associated cellular damage but have no effect on control cells and; secondarily that a high dose of SFA would be toxic to brain cells in both OGD and untreated control normal cells. We used primary brain cells extracted from the cortex of Long Evans rat, exposed them to an OGD environment to mimic PI, and treated the cells with various doses of SFA. We determined that SFA, at low doses, showed a protective effect by significantly preventing the death of brain cells in an OGD environment.

## Methodology

All animal work was approved by the Animal Care and Use Committee at the University of Alberta (AUP00000363). Long-Evans rats were purchased from Charles River (Montreal, PQ) and were kept on a 12 hour light/dark cycle with ad libitum access to food and water. Male and female rats were bred and pups delivered vaginally. The day of birth was determined to be postnatal day 1 (PD1).

### Isolation of cortical tissue

Rat cortical tissue was prepared from PD2 Long-Evan rats of both sexes. Brains were dissected and cortices were removed from meninges, isolated and transferred to a Petri dish containing calcium and magnesium free (CMF) Hank’s Balanced Salt Solution (HBSS) (Gibco, cat. no. 14170–112). Cortical tissues were enzymatically digested in 1mg/mL papain (Thermo Scientific, cat. no. 88285E) for 10 minutes at 37°C. DNase I (Millipore Sigma, cat. no. 11284932001) was added to the digestion mix in the last 5 minutes of incubation. Fetal Bovine Serum (FBS) (Gibco, cat. no. 12483–020) was added to stop the action of papain. Samples were centrifuged at 200g for 1 min and supernatant was aspirated. Cortices were triturated by pipetting 10 times with a glass Pasteur pipette.

### Isolation of neurons

The cell suspension was filtered through a 70 μm Nylon mesh cell strainer (ThermoFisher Scientific, cat. no. 22363547) with cell culture media containing Neurobasal-A medium (Gibco, Cat# 10888–022) supplemented with 2X B27 (Gibco, #17504–044), 1X N2 (Gibco, cat. no. 17502–048), 4X glutaMAX I (Gibco, cat. no. 35050–061), and 2X Antibiotic-Antimycotic (Gibco, cat. no. 15240–062). Neurons were plated on poly-D-lysine (Millipore Sigma, Cat. no. P7280) coated wells at a density of 3x10^5^cells/well in 24-well plates and 6x10^4^cells/well in 96-well plates. Media was changed 24 hours after plating, and every 3 days. Experiments started on day 7 in culture.

### Isolation of astrocytes

The cell suspension was filtered through a 70 μm Nylon mesh cell strainer with cell culture media containing Astrocyte medium—animal (ScienCell, cat. no. 1831) supplemented with 2X Antibiotic-Antimycotic, 1X Astrocyte growth supplement (AGS) (ScienCell, cat. no. 1882), and 2X FBS. Astrocytes were plated on poly-D-lysine coated wells at a density of 1.5x10^5^cells/well in 24-well plates and 3x10^4^cells/well in 96-well plates. Media is changed 24 hours after plating, and every 3 days. Experiments started on day 12 in culture.

### Isolation of combined culture (co-culture)

The cell suspension was filtered through a 70 μm Nylon mesh cell strainer with cell culture media containing Neurobasal-A medium, supplemented with 2X B27, 4X glutaMAX I, and 2X Antibiotic-Antimycotic. Cells were plated on poly-D-lysine coated wells at a density of 3x10^5^cells/well in 24-well plates and 6x10^4^cells/well in 96-well plates. Media was changed 24 hours after plating, and every 3 days. Experiments started on day 7 in culture.

### Oxygen and glucose deprivation (OGD)

The oxygen sensor (Pro Ox: 110, BioSpherix) for the hypoxia chamber is set at 0% oxygen level. Oxygen in the incubator is replaced by a mixture of gas that is 95% nitrogen and 5% carbon dioxide at 37°C. Regular growth media is removed and plates are washed with 1X Phosphate Buffer Saline (PBS) (HyClone, cat. no. SH3025601) to ensure normal media is not present. Glucose-free DMEM (Gibco, cat. no. A14430-01) is degassed for 10 minutes using a vacuum to remove the oxygen within the media, supplemented with Antibiotic-Antimycotic, and added to the cells during the hypoxic insult. Once the oxygen has been removed from both the incubator and the media, the plates of cells are then placed in the hypoxia chamber for the appropriate amount of time. Oxygen levels were monitored throughout with an oxygen sensor. At all times, oxygen levels were below 1%. Control plates are kept in normoxic conditions with regular media.

### 24 hour recovery

DMEM is replaced with regular growth media, and then the cells are placed in a normoxic incubator at 37°C with 5% carbon dioxide and 95% air, to recover for 24 hours.

### SFA treatment

R,S-Sulforaphane, dissolved in water, is obtained from LKT Laboratories, Inc. Cells were treated with SFA at different doses (0 μM– 200 μM). SFA is added to degassed DMEM for OGD and added to regular growth media for control plates and recovery.

### Preparation of cell lysates

Cells were washed with 1X PBS and then RIPA Lysis Buffer (Millipore Sigma, cat. no. 20–188) containing protease inhibitor (Millipore Sigma, cat. no. P8430) and phosphatase inhibitor (Millipore Sigma, cat. no. 524629) was added to lyse the cells and prevent protein degradation. Lysed cells were centrifuged at 4°C for 5 minutes at 12,000 rpm. The pellet was discarded and the supernatant collected for protein concentration determination.

### Immunoblotting

Bicinchoninic Acid Protein (BCA) Assay was used to quantify the amount of protein in each sample. Equivalent amounts of protein were resolved by 12.5% Tricine polyacrylmide gel electrophoresis. Proteins were then transferred onto polyvinylidenedifluoride (PVDF) membranes (Bio-Rad, cat. no. 1620177). After the transfer, the membranes were blocked in 5% Blocking Milk (Carnation) in TBS (Tris-Base, NaCl) and incubated with various primary antibodies at room temperature for 1 hour or overnight at 4°C. Membranes are then washed with TBS-T (TBS and Tween) then incubated with horseradish peroxidase conjugated secondary antibodies (GE Healthcare UK Limited) at room temperature for 1 hour. Western blots were visualized using Western Lightning® Plus-ECL (PerkinElmer, LAS Inc.) Antibodies to the following proteins were used for this study: Anti-Neuronal Specific Enolase (NSE) (Abcam, cat. co. ab53025), Anti-Glial Fibrillary Acidic Protein (GFAP) (Abcam, cat. no. ab7260), Anti-CD68 (Abcam, cat. no. ab31630), and Actin (Santa Cruz Biotechnology Inc).

### Live-dead assay

Following 24 hour recovery, live/dead assay (Thermofisher Scientific, cat. no. L3224) was used to determine percentage of cell death. Cells were stained with calcein-AM or ethidium homodimer-1 if they were alive or dead respectively and were left to incubate at room temperature for 10–15 minutes. Cell counting was done using a high content analysis system, MetaXpress XLS (Molecular Devices, San Jose, CA, USA). To determine the LD50, Cell Death (CD) % was normalized to control plates: 100 x [(sample CD—control CD) x (100 –control CD)].

### AlamarBlue cytotoxicity assay

The relative cytotoxicity of different concentrations of SFA was established using an AlamarBlue (AB) assay. Using the REDOX indicator resazurin (oxidized form), it is possible to spectrophotometrically measure the cellular proliferation. Resazurin is blue and non-fluorescent, whereas resorufin (reduced form) is red/pink and highly fluorescent. Thus, measuring the changes in the fluorescence of the dye in the intracellular environment, modifications in the number of metabolic active cells can be detected. The AB assay was carried out according to manufacturer’s instructions. Cells were incubated with different concentrations of SFA (0 μM– 200 μM) using untreated cells (0 μM SFA) as control cells, after 24 hr of incubation AB solution (10% [v/v] solution of AB dye) was added into 100μl of complete media to each well. Wells containing only the AB solution/media without cells was used as the blank. Following 2 h incubation, AB fluorescence was quantified at the respective excitation and emission wavelength of 540 and 595 nm respectively, using a LumIstar Omega reader. Viability % was normalized: (sample relative fluorescent unit (RFU)–Blank) X 100/(Untreated cells RFU- Blank), using the untreated cells.

### Immunofluorescence

Cells were washed with 1X PBS and treated with 4% paraformaldehyde (PFA) (Thermofisher Scientific, cat. no. 15710) for 15 mins to fix cells. Cells were washed again with 1X PBS, followed by treatment with 0.2% 100X Triton (Thermofisher Scientific, Fisher BioReagents cat. no. BP151500) in 1X PBS for 10 minutes to permeabilize cells. After washing with 1X PBS again, wells were blocked with 2% bovine serum albumin (BSA) (Thermofisher Scientific, Fisher BioReagents cat. no. BP1600-100) in 1X PBS at room temperature for 1 hour or overnight at 4°C, and then incubated with various primary antibodies at 4°C overnight. Wells were washed with 1X PBS, and then incubated with secondary immunofluorescent (IF) antibodies at room temperature for 1 hour. Cell characterization of co-cultures was established via IF cell images visualized using a laser scanning confocal microscope (Zeiss LSM710) and quantified using a high content analysis system, MetaXpress XLS. DAPI was used as a cell staining control and total cell count. Antibodies to the following proteins were used for this study: Anti-NSE, Anti-GFAP, Anti-CD68.

### Statistical analysis

GraphPad Prism 5 computer software was used. Data were from at least 3 experiments with a minimum of triplicates per experiment. Values were expressed as means ± SE. Statistical significance was analyzed using one-way ANOVA with Dunnett’s Multiple Comparisons Test. Significance is indicated by *(*p<*0.05), **(*p<*0.01), ***(*p<*0.001), or ****(*p<*0.0001).

## Results

### Cell culture purity

**Fig 1:**
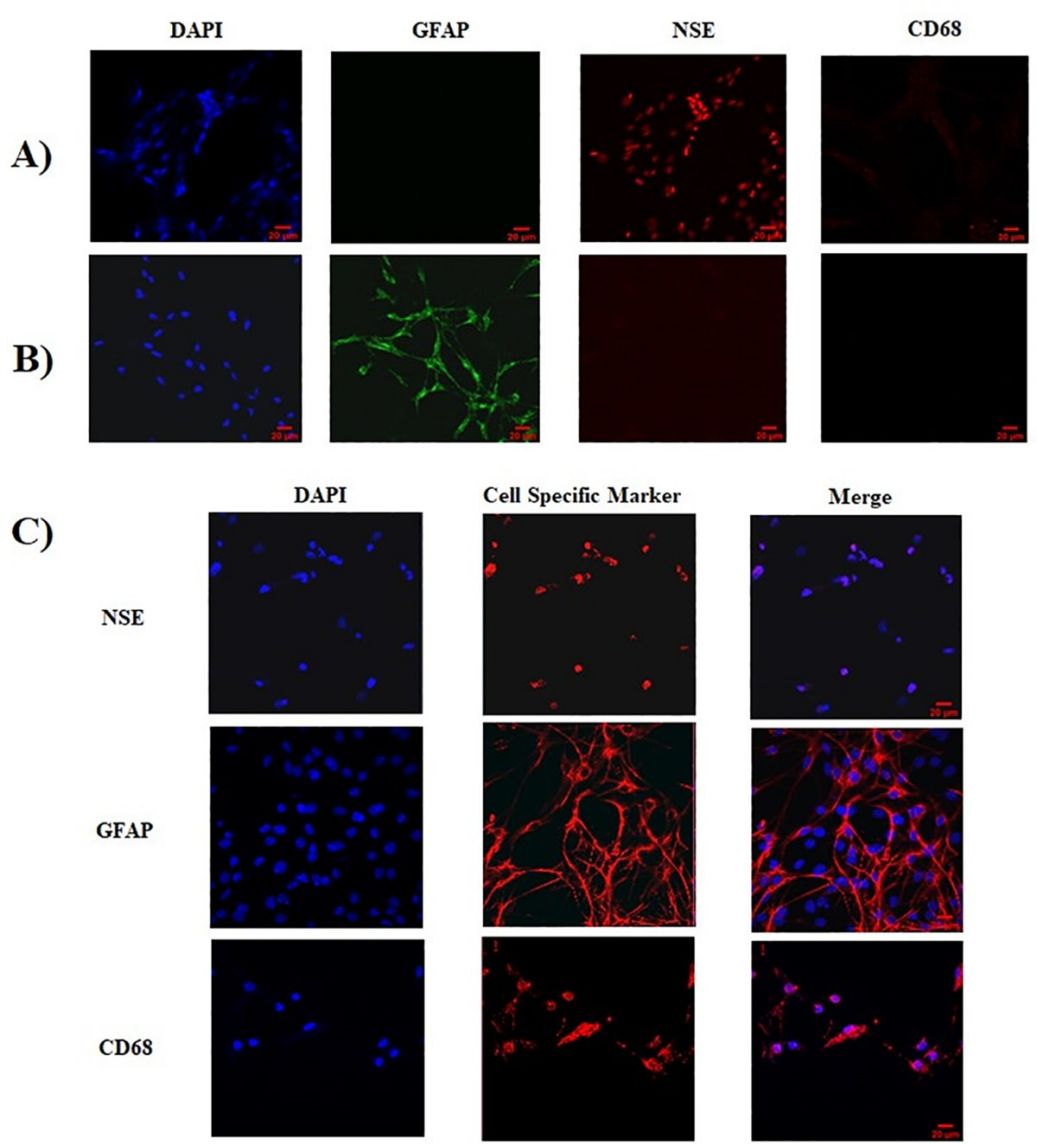
Immunofluorescence analysis to confirm the characterization of control cortical brain cell cultures. DAPI is a staining control and represents the nuclei of all cells. Cell specific markers used were: NSE for neurons, GFAP for astrocytes, and CD68 for microglia. **A)** Neuron culture **B)** Astrocyte culture, **C)** Co-culture. Scale shown is 20 μm.

Immunofluorescence (IF) images show the characterization of neuronal cultures, astrocyte cultures, and co-cultures. Neuronal cultures show enhanced staining for NSE protein (Fig 1A) compared to a lower detection of astrocytes or microglia. Astrocyte cultures show enhanced staining for GFAP protein (Fig 1B) compared to a lower detection of neurons or microglia. Co-Cultures (Fig 1C) show the presence of all cell types: neurons, astrocytes and microglia. Cell nuclei are stained with DAPI.

### LD50 determination

**Fig 2:**
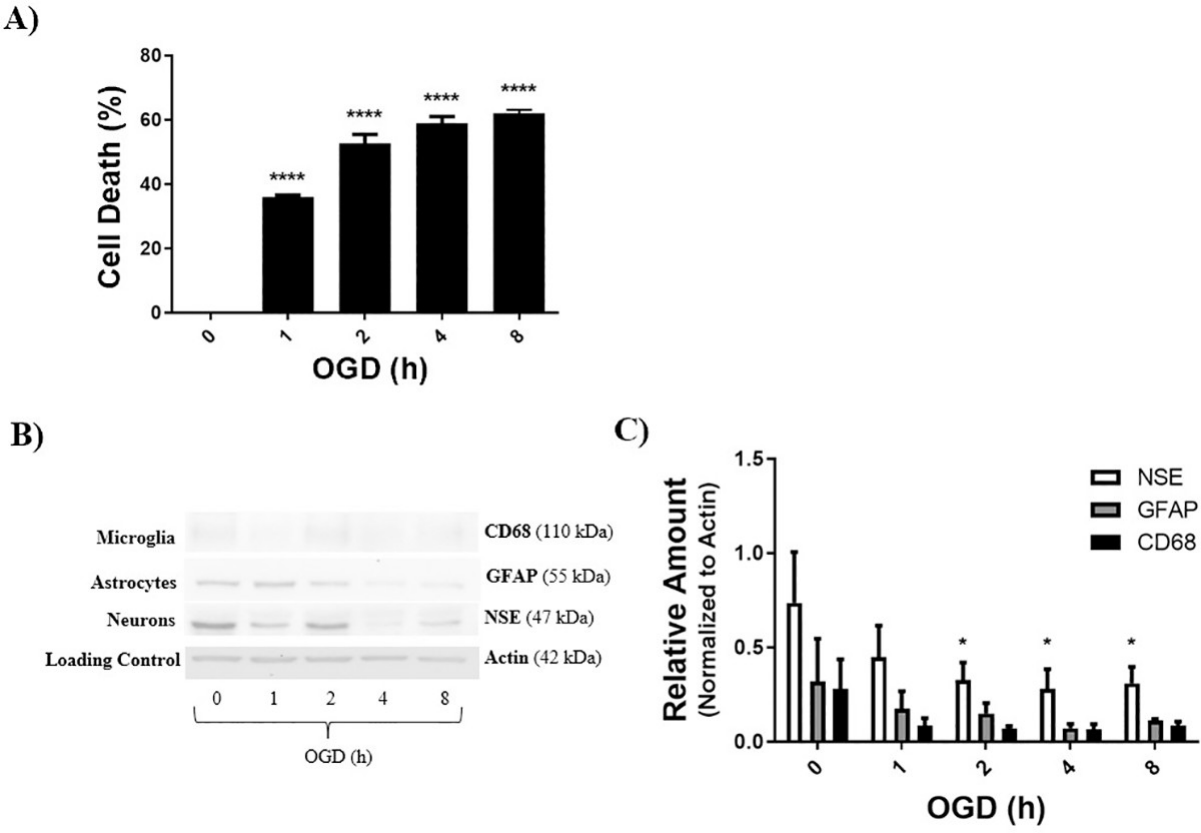
LD50 for neuronal cell culture was determined using a live dead assay, and further analyzed by western blot and densitometry. **A)** Neuronal cell cultures achieved an equal to or above 50% cell death at 2 hours OGD, data was normalized to control (0h OGD). **B, C)** Actin represents the loading control. Cell specific markers used were: NSE for neurons, GFAP for astrocytes, and CD68 for microglia. Data represented as mean±SEM, n≥3. One way ANOVA, and Dunnett’s Multiple Comparison Test was completed; *p<0.05, **p<0.01, ***p<0.001, ****p<0.001, compared to 0 hour OGD control.

**Fig 3:**
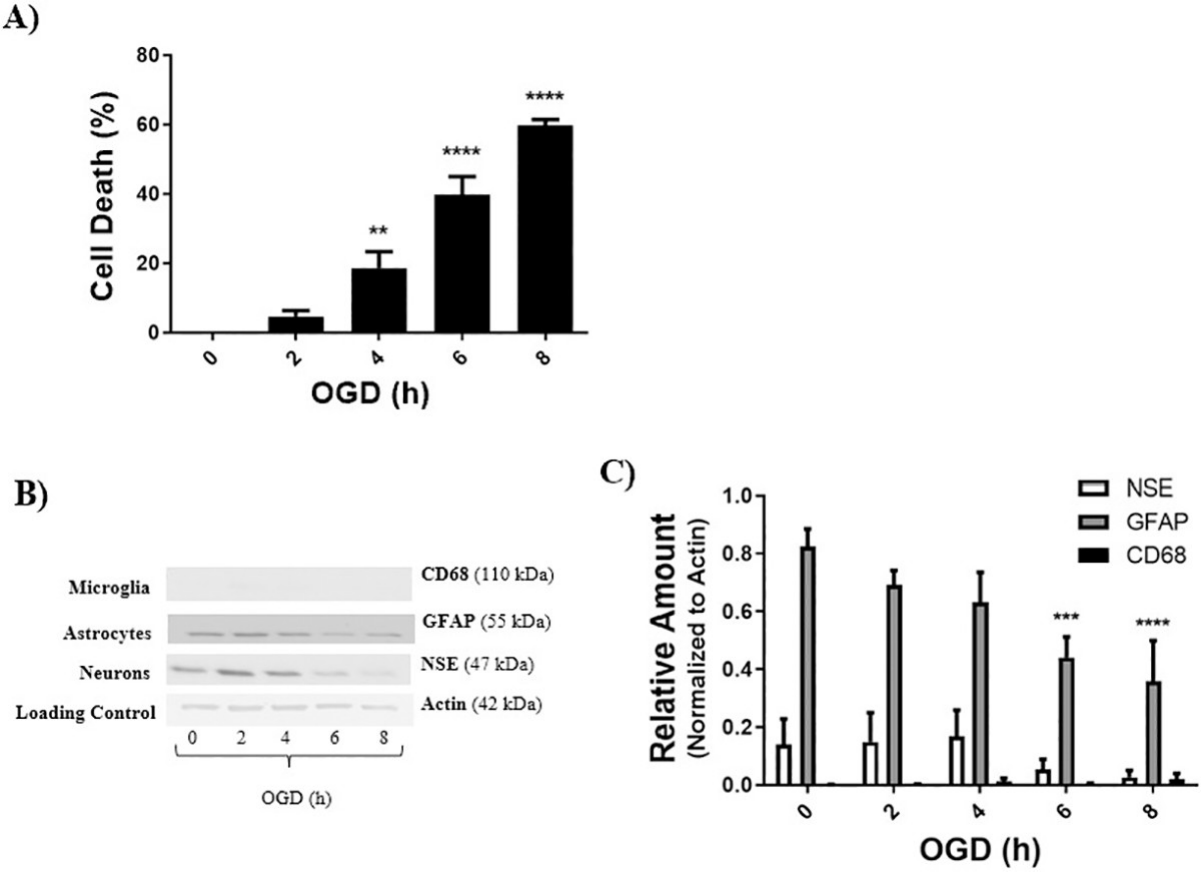
LD50 for astrocyte cell culture was determined using a live dead assay, and further analyzed by western blot and densitometry. **A)** Astrocyte cell cultures achieved an equal to or above 50% cell death at 8 hours OGD, data was normalized to control (0h OGD). **B, C)** Actin represents the loading control. Cell specific markers used were: NSE for neurons, GFAP for astrocytes, and CD68 for microglia. Data represented as mean±SEM, n≥3. One way ANOVA, and Dunnett’s Multiple Comparison Test was completed; *p<0.05, **p<0.01, ***p<0.001, ****p<0.001, compared to 0 hour OGD control.

**Fig 4:**
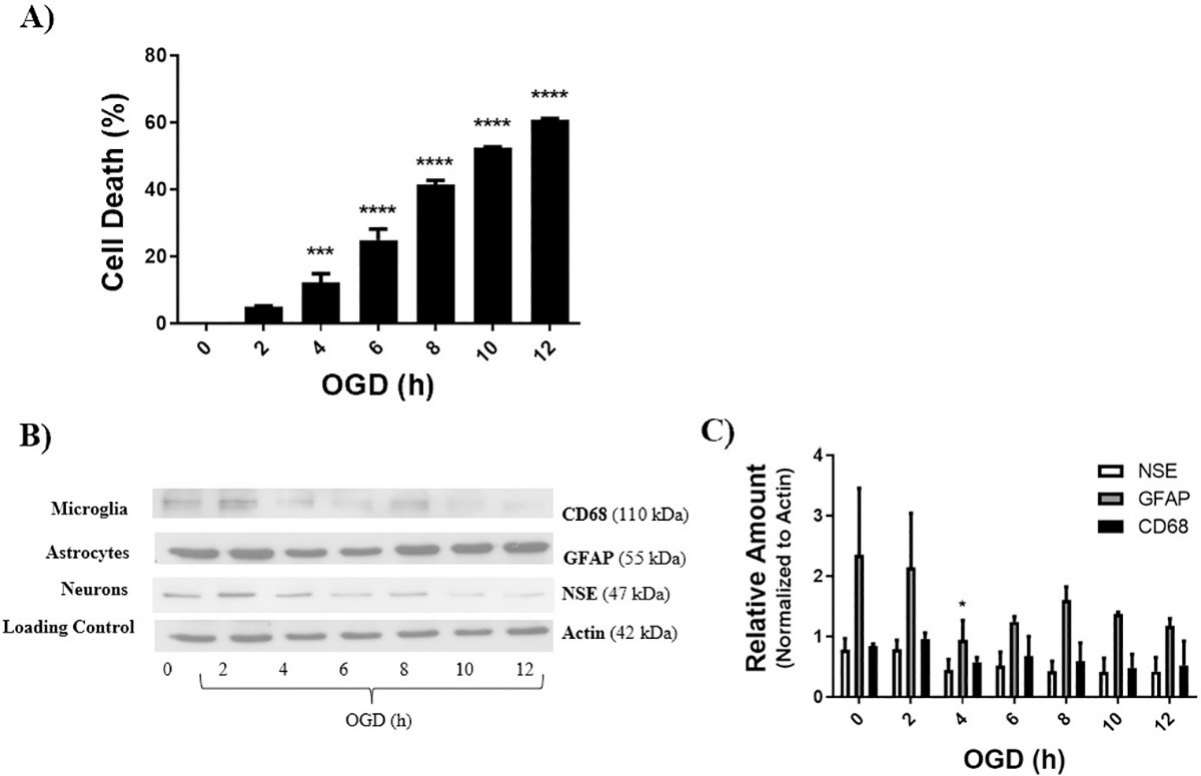
LD50 for co-culture was determined using a live dead assay, and further analyzed by western blot and densitometry. **A)** Co-cultures achieved an equal to or above 50% cell death at 10 hours OGD, data was normalized to control (0h OGD). **B, C)** Actin represents the loading control. Cell specific markers used were: NSE for neurons, GFAP for astrocytes, and CD68 for microglia. Data represented as mean±SEM, n≥3. One way ANOVA, and Dunnett’s Multiple Comparison Test was completed; *p<0.05, **p<0.01, ***p<0.001, ****p<0.001, compared to 0 hour OGD control.

LD50 was defined as the duration of OGD that achieved equal to or above 50% cell death. The LD50 for each cell culture was determined by a Live/Dead assay, and cell death for each OGD duration was normalized to control (0h OGD). Increase in OGD duration showed significant cell death in neurons at 1, 2, 4, and 8 hours, compared to 0 hour OGD control (Fig 2A). LD50 was determined to be 2 hours for neurons (Fig 2A). Western blot analysis of the neuronal culture for the neuronal marker NSE shows that cellular levels of NSE are consistently greater than GFAP and CD68, and NSE levels decrease significantly with increasing duration of OGD (Fig 2B and 2C). Increase in OGD duration showed significant cell death in astrocytes at 6 and 8 hours, compared to 0 hour OGD control (Fig 3A). LD50 was determined to be 8 hours for astrocytes (Fig 3A). Western blot analysis of the astrocyte culture for the astrocyte marker GFAP shows that cellular levels of GFAP are consistently greater than NSE and CD68, and GFAP levels decrease significantly with increasing duration of OGD (Fig 3B and 3C). Increase in OGD duration displayed significant cell death in co-cultures at ≥ 4 hours, compared to 0 hour OGD control (Fig 4A). LD50 for the co-culture of brain cells was determined to be 10 hours (Fig 4A). Fig 4B shows the relative cellular levels of the different cell markers with time of OGD, with a significant decrease in neurons being evident at 4 hours OGD. CD68, a biomarker for microglia, are either undetectable (Figs 3B and 4B) in the astrocyte and co-cultures or present in very small quantities (Fig 2B) in the neuronal culture.

### SFA dose response in OGD cultures

**Fig 5:**
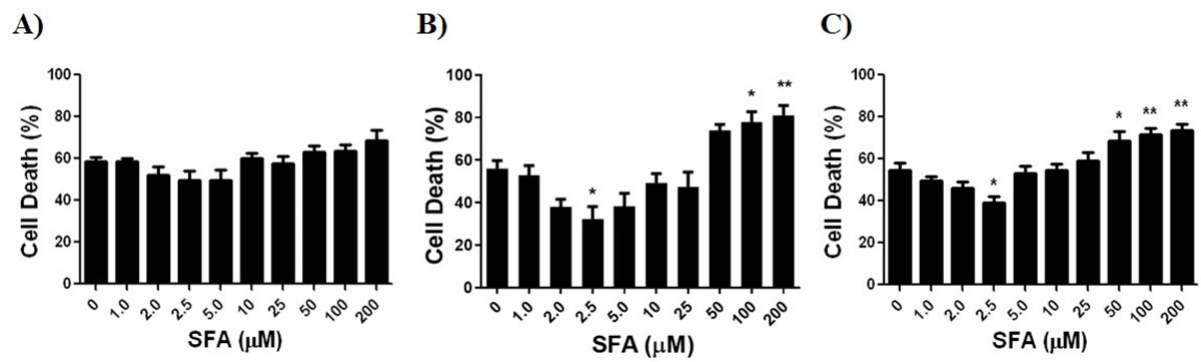
SFA dose response for each brain cell-type culture in an OGD environment was determined using a live dead assay at previously determined LD50. **A)** Neuronal cultures at LD50 of 2 hours OGD, no significant protection or toxicity, **B)** Astrocyte cultures at LD50 of 8 hours OGD, protection of SFA at 2.5 μM, significant toxicity of SFA ≥ 100 μM, **C)** Co-cultures at LD50 of 10 hours OGD, protection of SFA at 2.5 μM and toxicity of SFA ≥ 50 μM. Data represented as Mean±SEM, n≥3, One way ANOVA, and Dunnett’s Multiple Comparison Test was completed for all cultures; *p<0.05, **p<0.01, ***p<0.001, ****p<0.001, compared to respective controls (0 μM SFA).

OGD cultures at the previously determined time of LD50 were treated with SFA. The protective dose of SFA was determined as that which resulted in a significantly lower cell death due to OGD compared to control cell death (0 μM SFA). Cytotoxic doses of SFA was determined as the dose of SFA that resulted in a significantly higher cell death due to OGD compared to control cell death. Fig 5A shows that with increasing doses of SFA there was initially a decrease in cell death at 2 hours OGD treatment of neurons, most prominently at 2.5 μM SFA, although not significant. In the presence of 2.5 μM SFA, there was a significant decrease in cell death following 8 hours of OGD treatment in astrocytes and 10 hours OGD treatment of co-cultures (Fig 5B & 5C). Higher doses of SFA (≥ 100 μM), presented a significant toxic effect on astrocytes (Fig 5B), and OGD treated co-cultures exhibited a significant toxic effect in the presence of ≥ 50 μM SFA (Fig 5C).

### SFA dose response in control cultures

**Fig 6:**
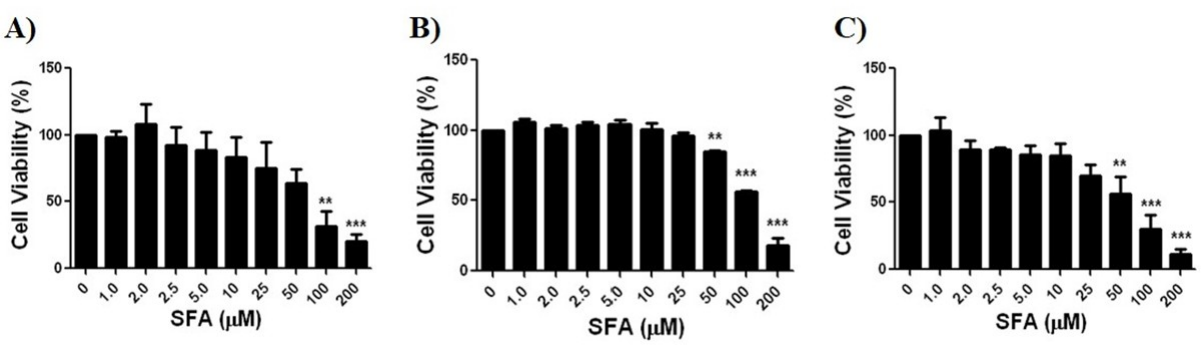
SFA dose response for each brain cell-type control culture was determined using an AlamarBlue assay. **A)** Neuronal cultures showed toxicity of SFA ≥ 100 μM, **B)** Astrocyte cultures showed toxicity of SFA ≥ 50 μM, **C)** Co-cultures showed toxicity of SFA ≥ 50 μM. Data represented as Mean±SEM, n≥3, One way ANOVA, and Dunnette’s Multiple Comparison Test was completed for all cultures; *p<0.05, **p<0.01, ***p<0.001, ****p<0.001, compared to respective controls (0 μM SFA).

In order to determine potential cytotoxic effects of SFA on brain cells in a normoxic/normal glucose environment, control cultures (no OGD) were treated with SFA, again at varying doses. Cytotoxic doses of SFA were determined as the dose of SFA that resulted in a significantly lower cell viability compared to 0 μM SFA. Cell viability, determined using AlamarBlue assay, shows that SFA had no effect on the viability of neurons, astrocytes or co-cultures at low concentrations (Fig 6A–6C). SFA at doses ≥ 100 μM were significantly toxic to neuronal cell cultures (Fig 6A). Both astrocyte and co-cultures showed significant cell death at SFA doses ≥ 50 μM (Fig 6B & 6C).

Live/Dead assay was also used to show cytotoxicity of SFA in control cultures. Cytotoxic doses of SFA were determined to be the dose of SFA that resulted in a significantly higher cell death compared to 0 μM SFA. SFA had no effect on the viability of neurons, astrocytes or co-cultures at lower concentrations (S1A–S1C Fig). SFA was not significantly toxic to neuronal cell cultures, although a gradual increase in cell death is seen as SFA dose increases (S1A Fig). Both astrocyte and co-cultures showed significant cell death at SFA doses ≥ 50 μM (S1B Fig) and ≥ 200 μM (S1C Fig), respectively.

## Discussion

This study demonstrates the importance of dose dependent effects of SFA in different cortical brain cells. We show that low doses of SFA are protective in an OGD environment to PD2 cortical brain cell cultures and are safe in control cultures. On the other hand, high doses of SFA are cytotoxic under OGD and control normal oxygen and glucose conditions. Preventing cell death is key to the prevention of neurodevelopmental disabilities during pregnancy, as brain cell injury can lead to impaired cell survival, migration and connectivity that result in impaired motor, sensory, cognitive and behavioral functions. Conventional therapies, used at time of birth, have not been shown to be effective in reversing or repairing the brain damage that has already occurred. Hypothermia administered within a few hours after birth is only effective for injuries that occur during delivery, successfully slows down brain metabolism and reduces mortality, but has not been definitively shown to reduce CP incidence or severity [3]. Hypothermia treatment does not salvage brain cells that are already injured or dead from an insult [1]. Moreover, it will only treat that small percentage of aetiologies that result from injury around the time of birth (10–20%). Further, when used as a therapeutic after injury, there has been contradictory evidence of SFA being effective in animal models [24]. It is important to note that our previous studies aimed to determine whether SFA could be used as a prophylactic preventative regimen by pregnant women to target and reduce oxidative stress induced injury to fetal brain cells before it leads to cell death. The current study aims to determine the range of SFA dosing that is within safety and toxicity.

Although this study focuses primarily on the effect of SFA on cortical brain cells, perinatal brain injury can take place, often simultaneously, in both gray and white matter [7–9]. A common cause for perinatal brain injury is HI induced rise in ROSs which lead to cell death through activation of apoptotic receptors or proteins, and mitochondrial dysfunction. ROS, produced by microglia and leukocytes, are a defense mechanism activated upon injury, which can cause death or damage of surrounding brain cells including neurons, astrocytes, and oligodendrocytes [21,25].

Gray matter injury is mainly due to cell death or functional compromise of neurons of the cortex, thalamus, and hippocampus; this is often also accompanied by damage to astrocytes [8,9]. Astrocytes play a dominant role in the protection of neurons by increasing anti-oxidative enzymes through the Nrf2/ARE pathway which can neutralize ROS to prevent neuronal cell damage [25–27]. Neurons also use this same protective mechanism, though it is less efficient than in astrocytes, as neurons are inadequate up-regulators of anti-oxidants and have a low resting glutathione levels, which is central for redox homeostasis of the cell [2,21,25]. This paradigm is supported by our data which showed that the protective effect of SFA on OGD neurons (Fig 5A), was not as effective compared to the protective effect of SFA on OGD astrocytes (Fig 5B); the difference in cell death decrease from 0 μM to 2.5 μM was over 2 fold greater in astrocytes compared to neurons. However, the most significant protective effect of SFA was observed in the co-cultures (Fig 5C), which is the brain cell culture most representative of brain tissue. We postulate that the high protective efficacy of SFA in the co-cultures is due to the anti-oxidative enzymes released by the astrocytes through the activation of the Nrf2/ARE pathway, upon OGD insult, effectively neutralizing ROS and preventing cell damage.

The co-culture is most representative of the brain as it consists of all brain cell types extracted from the PD2 brain tissue. This would also explain why the LD50 time for co-cultures (Fig 4A) was higher compared to the LD50 time for neurons (Fig 2A) and astrocytes (Fig 3A). The co-culture contains astrocytes which function to protect the neurons, as well as microglia which activate immune responses. Additionally, although not investigated in this study, there also may be the presence of oligodendrocytes in the co-culture which also protect the neurons through myelination. Neuron cells are mainly effect by HI via two phases [28]. Initially neuron death can occur due to lack of overall energy from reduced adenosine triphosphate (ATP). Reduced ATP levels lead to an improper functioning ion channels and cell membrane depolarization and an accumulation of extracellular glutamate. Accumulation of intracellular lactate also results in an increase of ROS which leads to cell swelling and necrosis [28]. A Secondary energy failure can occur after a recovered blood flow and results in mainly apoptosis through excitotoxicity, oxidative stress, inflammation, and other factors. This second phase of neuron cell death contributes to majority of cell death in PBI [28]. Upon damage, dying or dead cells in the brain release cellular debris which prompts astrocytes and microglia cells to activate an immune response [28]. Upon injury (OGD), microglia are activated and aid in exacerbating or limiting injury through phagocytosis of cellular debris, produce pro-inflammatory cytokines and ROS scavenging receptors [28,29]. Excitotoxicity, oxidative stress, and cytokine signalling also contribute to damage of oligodendrocytes. Excitotoxicty against oligodendrocytes works also by extracellular glutamate accumulation and results in an increase of ROSs causing oxidative stress. These processes are detrimental to the brain; by damaging oligodendrocytes they specifically reduce or attenuate the myelination process, thereby creating vulnerable neurons [2,7,9,30,31]. With all of these cellular constituents present in the co-culture, it is more likely to thrive compared to isolated neuron or astrocyte cultures; and their response to effects of SFA is more representative of what an *in vivo* response may look like.

The Nrf2/ARE mechanism and its activation by SFA have been well established [13,15,16,18–20]. The significance of using SFA as an inducer of the Nrf2/ARE pathway is that it upregulates many phase II anti-oxidant enzymes including glutathione peroxidase (GPX), glutathione S-transferase (GST), heme oxygenase 1 (HO-1), NADPH quinine oxidoreductase 1 (NQO-1) among others [21,32]. Phase II anti-oxidant enzymes can directly remove ROSs and reduce oxidative stress [21]. An important anti-oxidant enzyme that is many times indirectly enhanced by SFA is GPX, a key component of the glutathione dependent anti-oxidant system, vitally important in maintaining endogenous redox homeostasis [2,31]. GPX is involved in the conversion of reduced glutathione (GSH) to oxidized glutathione (GSSG), by an oxidation process which also includes neutralizing ROSs [2,25]. However, the immature brain has low levels of GPX and therefore a reduced endogenous ability to scavenge ROS [31]. Our study shows that SFA efficiently and significantly prevents cell death in an HI environment. Since SFA can cross the blood-brain and placental barrier, it is possible that SFA may prevent cell death in the fetal brain under oxidative stress through upregulation of oxidative enzyme GPX via activation of the Nrf2/ARE pathway [21,31]. However, this interaction needs to be further studied as SFA may not interact with GPX in all oxidative stress circumstances or cells.

Our study shows that SFA is safe and protective of brain cells in cultures at low doses (≤ 2.5 μM) while it is toxic at high doses. SFA has previously been shown to be a hormetic compound which exhibits a biphasic dose response. While the protective effect of SFA has been shown to work prominently through the Nrf2/ARE pathway, the toxic effect of SFA is impacted by various other mechanisms that result in cell death [33–35]. We have also shown that ≥ 50 μM SFA dose has been toxic in both OGD (Fig 5) and control cell cultures (Fig 6), and these mechanisms of toxicity can include inhibiting histone deacetylase [34,35], and inhibiting upregulation of tumor suppressor proteins [33]. Our observation that a low dose of 2.5 μM SFA is protective in brain cell cultures exposed to OGD (Fig 5) is supported by other studies that demonstrate a protective effect of SFA at doses ≤ 2.5 μM [18,36–38]. Kraft et al. showed that pre-treatment of cortical astrocytes and neuronal cells for 48 hours with 2.5 μM SFA resulted in an increase of ARE dependent genes including total GSH levels, upon H_2_O_2_ induced oxidative stress [18]. In addition, 1 μM SFA was reported to upregulated Nrf2 in neural crest cells and ameliorate the effects of EtOH induced apoptosis in animal models of fetal alcohol syndrome disorder [36–38].

From the literature it is evident that there may be a shift in SFA mechanism from protective to toxic in a dose dependant manner. At very low doses near 2.5 μM, SFA is cytoprotective as shown in this study and previously reported in the literature [18,36–38]. At high doses, ≥2.5 μM, SFA is cytotoxic. It seems that doses within this range may be protective and/or toxic based on culture conditions, creating a shift in metabolic processes. A significant governing body of homeostasis in a cell is the adenoside monophosphate-activated protein kinase (AMPK) pathway which may play a role in the protective and toxic effects of SFA [39,40]. Many studies have shown a relationship between AMPK and SFA and pro-apoptotic or anti-apoptotic effects [39,40]. SFA has been used to activate AMPK in pancreatic cancer cells in a hypoglycemic environment to inhibit proliferation and promote apoptosis [39]. Alternatively, SFA has been shown to protect neuronal cells from prion protein induced apoptosis by increasing AMPK phosphorylation [40].

The in vitro cell culture model used in this study has limitations. All cell cultures were prepared at P2. Clearly, in-vitro cell culture studies do not exactly reflect an in-vivo full tissue environment. However, it is important to reflect on the goals of our study, which were to: 1. Determine if there is a differential cellular effect of SFA on individual brain cells, and 2. Determine the range of safety vs toxicity of SFA, given the paradoxical benefits of SFA in neuroprotection and cancer therapy. This latter goal is especially important given our laboratory findings of the benefit of SFA as a dietary supplement during pregnancy for the prevention of perinatal brain injury and childhood disability. In this regard, the fetus is a rapidly developing organism which has characteristics of both cell injury (PI) and cell division (normal development). Hence, understanding the boundaries of dosing is extremely important. Finally, our co-cultures were done in order to best assimilate the combination of cells found in the brain, as well as the extracellular environment of the cells.

In conclusion, we have determined that low doses of SFA in neuronal, astrocyte, and co-cultures is neuroprotective in an OGD environment, and safe in control cultures. High doses of SFA are toxic in all cultures in both OGD and control environments. It is important to note the difference between effective low protective dosing and high toxic dosing of SFA are at least a 10-fold difference which alludes to the safety in using SFA as a preventative approach in a perinatal setting. The Nrf2/SFA mechanism has been extensively research and is available in literature; in this study we extrapolated knowledge from the literature to explain effects of SFA in our neuron, astrocyte, and co-cultures [13,15,16,18–20]. Future experiments will include determining mechanistic effects between SFA, Nrf2, ARE, cytokines, ROSs, and ROS scavenging agents such as GPX, through protein identification, quantification, and enzyme assays. These experiments will be completed *in vitro* using mammalian cultures from this study; and mechanistic effects in each cell type along with co-cultures from this project will be investigated. Next steps will involve investigating effects of SFA during pregnancy on the potential for teratogenicity or organ damage in preclinical animal models; a step required to determine a safe and effective dose that can be translated to human pregnancy clinical trials.

## Supporting information

S1 Fig SFA dose response for each brain cell-type control culture was determined using a LiveDead assay. **A)** Neuronal cultures did not show significant toxicity, **B)** Astrocyte cultures showed toxicity of SFA ≥ 50 μM, **C)** Co-cultures showed toxicity of SFA ≥ 200 μM. Data represented as Mean±SEM, n≥3, One way ANOVA, and Dunnett’s Multiple Comparison Test was completed for all cultures; *p<0.05, **p<0.01, ***p<0.001, ****p<0.001, compared to respective controls (0 μM SFA).
(TIF)

S1 Raw images (PDF)
