# Supplementary figures and images for: Sulforaphane (SFA) protects neuronal cells from oxygen & glucose deprivation (OGD)
Source: PLoS One. 2021 Mar 18;16(3):e0248777. doi: 10.1371/journal.pone.0248777 (PMC7971874; doi:10.1371/journal.pone.0248777)

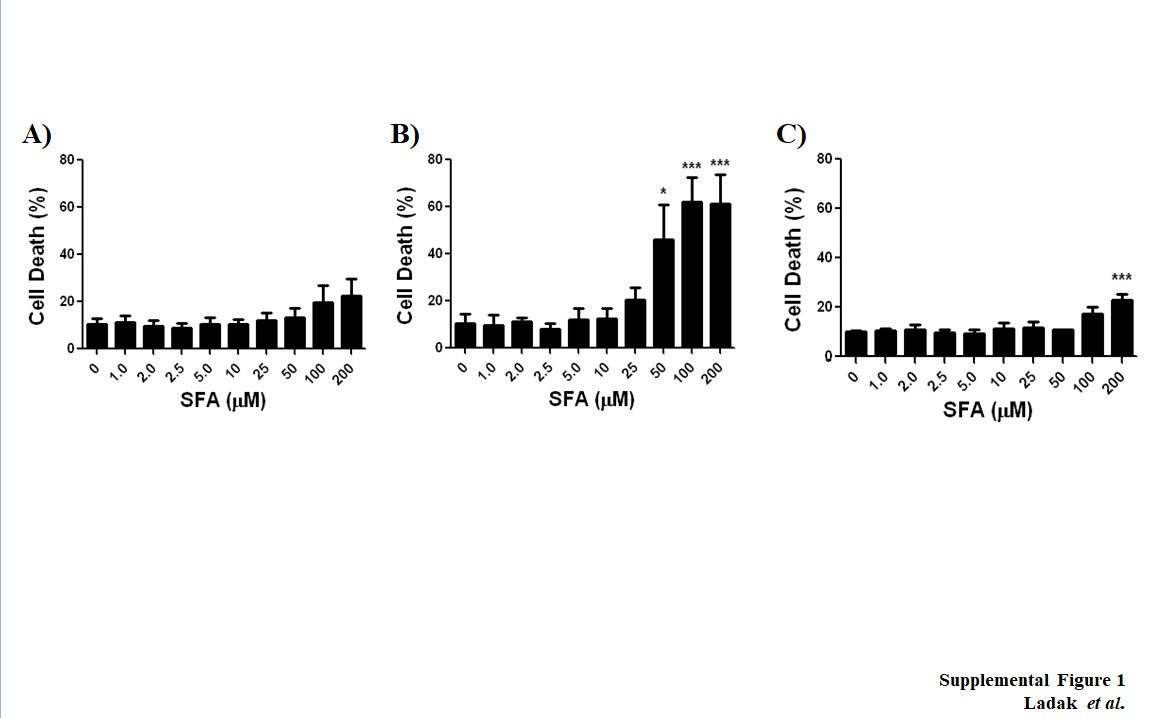

Supplement: S1 Fig — A) Neuronal cultures did not show significant toxicity, B) Astrocyte cultures showed toxicity of SFA ≥ 50 μM, C) Co-cultures showed toxicity of SFA ≥ 200 μM. Data represented as Mean±SEM, n≥3, One way ANOVA, and Dunnett’s Multiple Comparison Test was completed for all cultures; *p<0.05, **p<0.01, ***p<0.001, ****p<0.001, compared to respective controls (0 μM SFA). (TIF) [file pone.0248777.s001.tif]

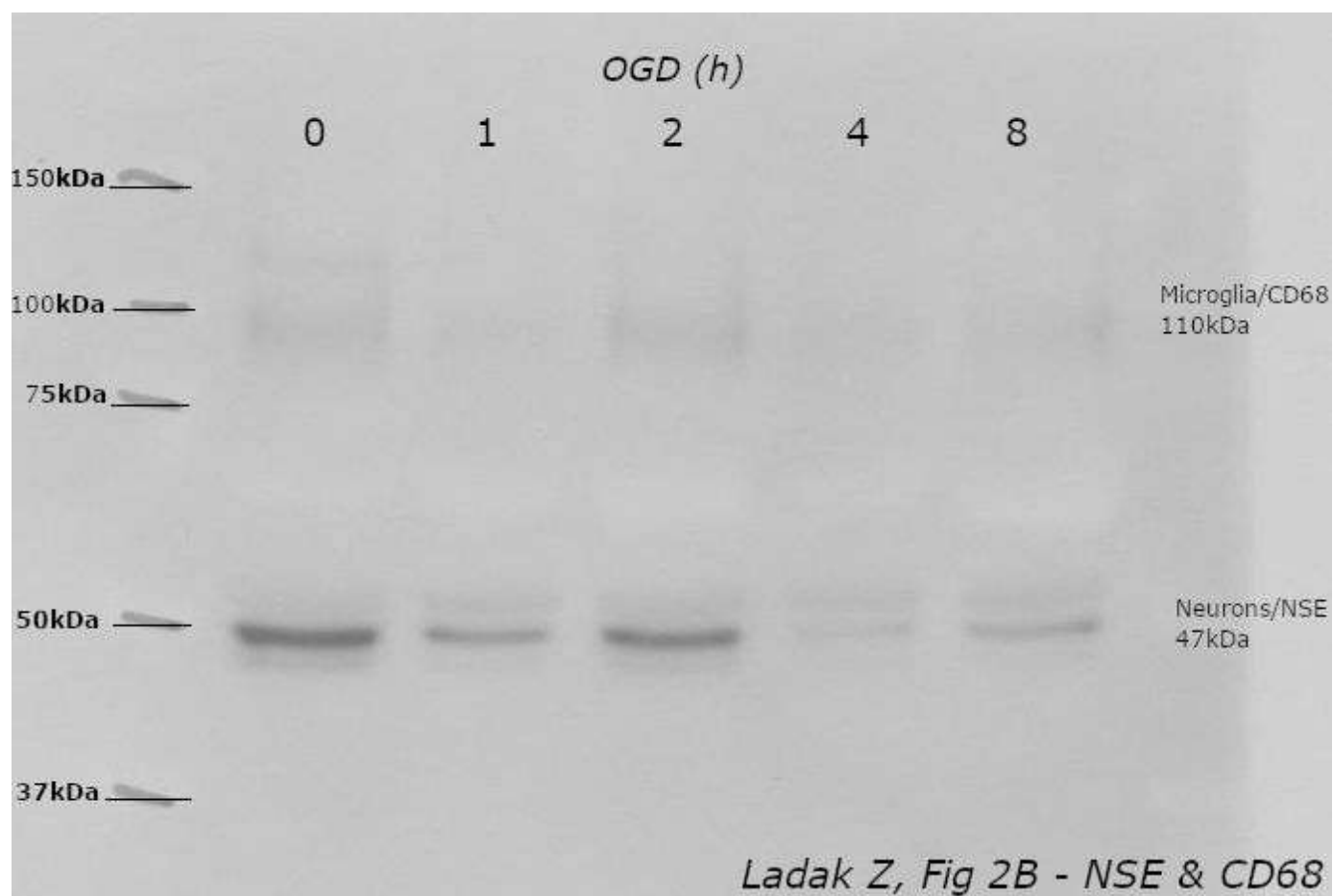

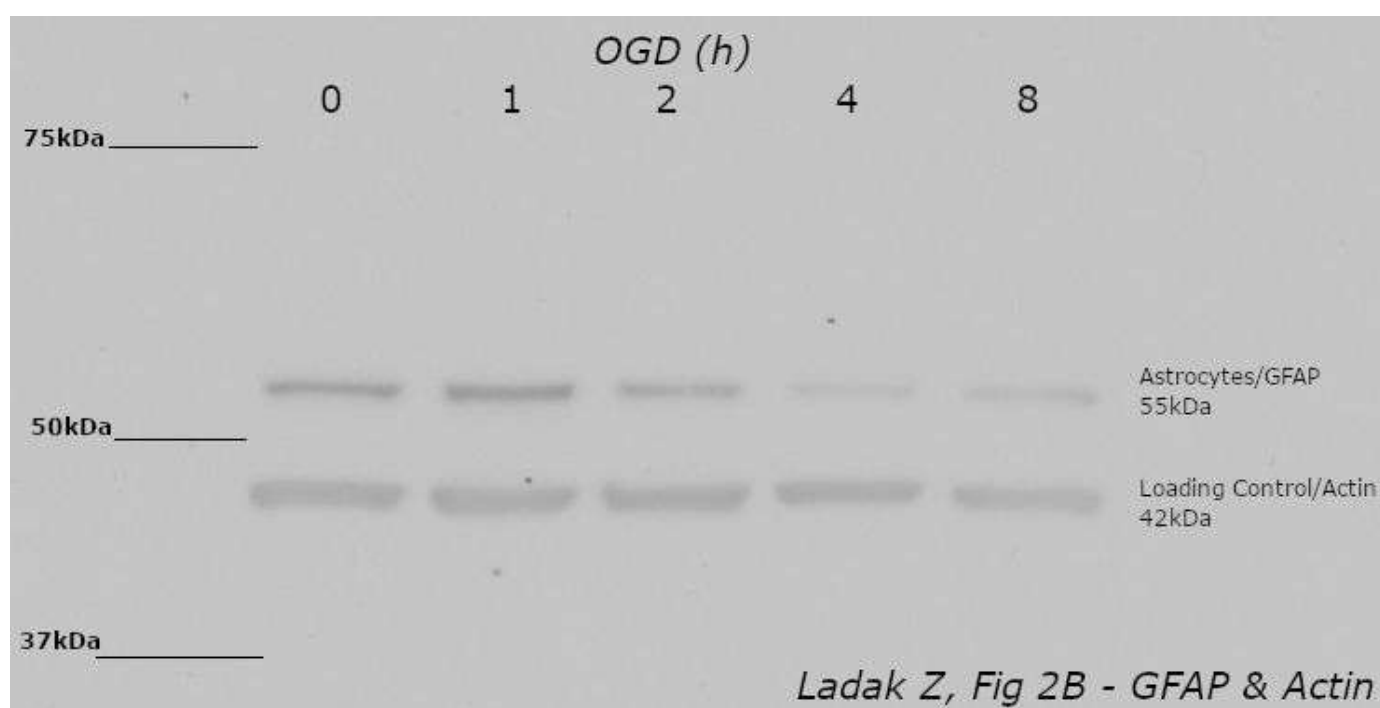

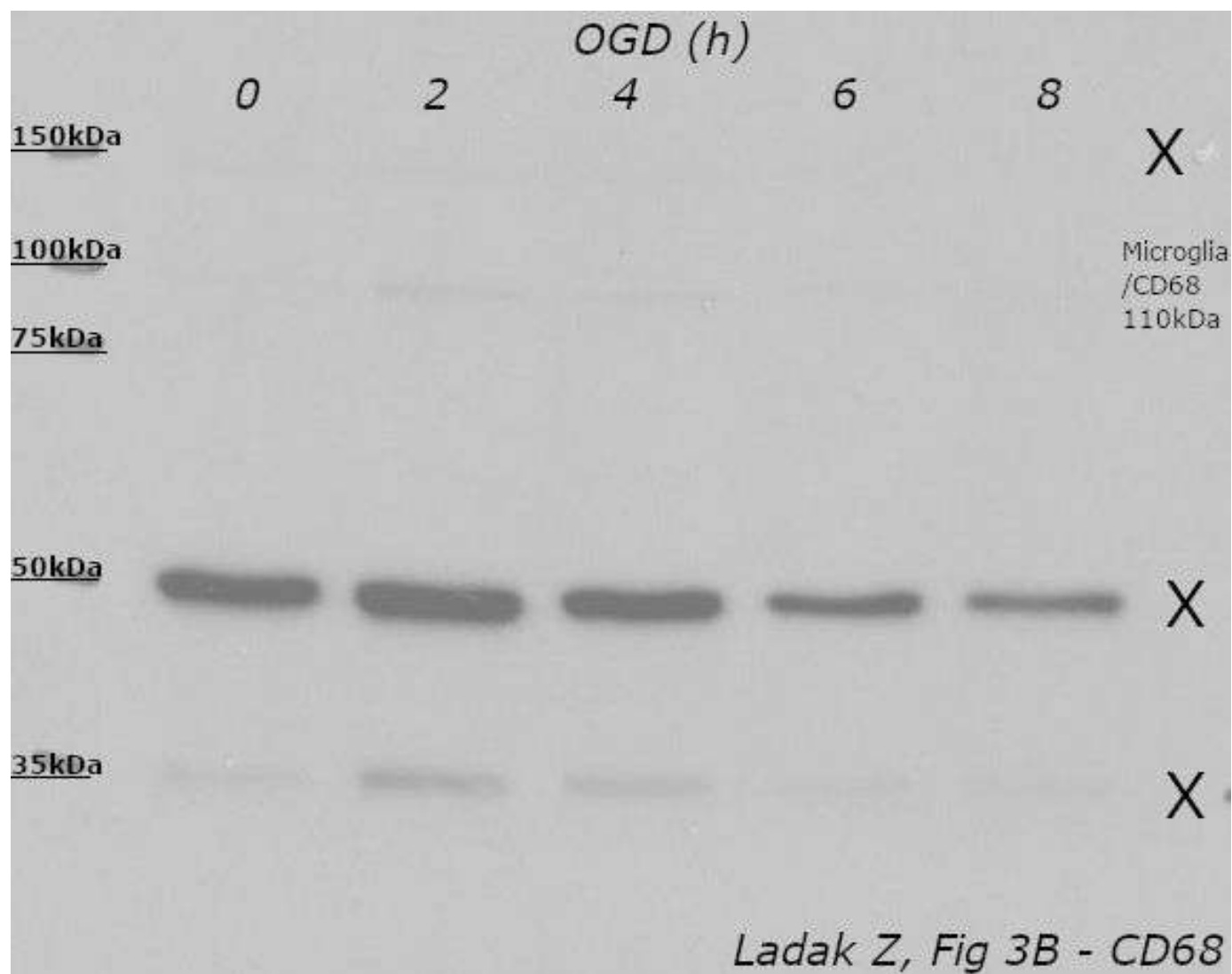

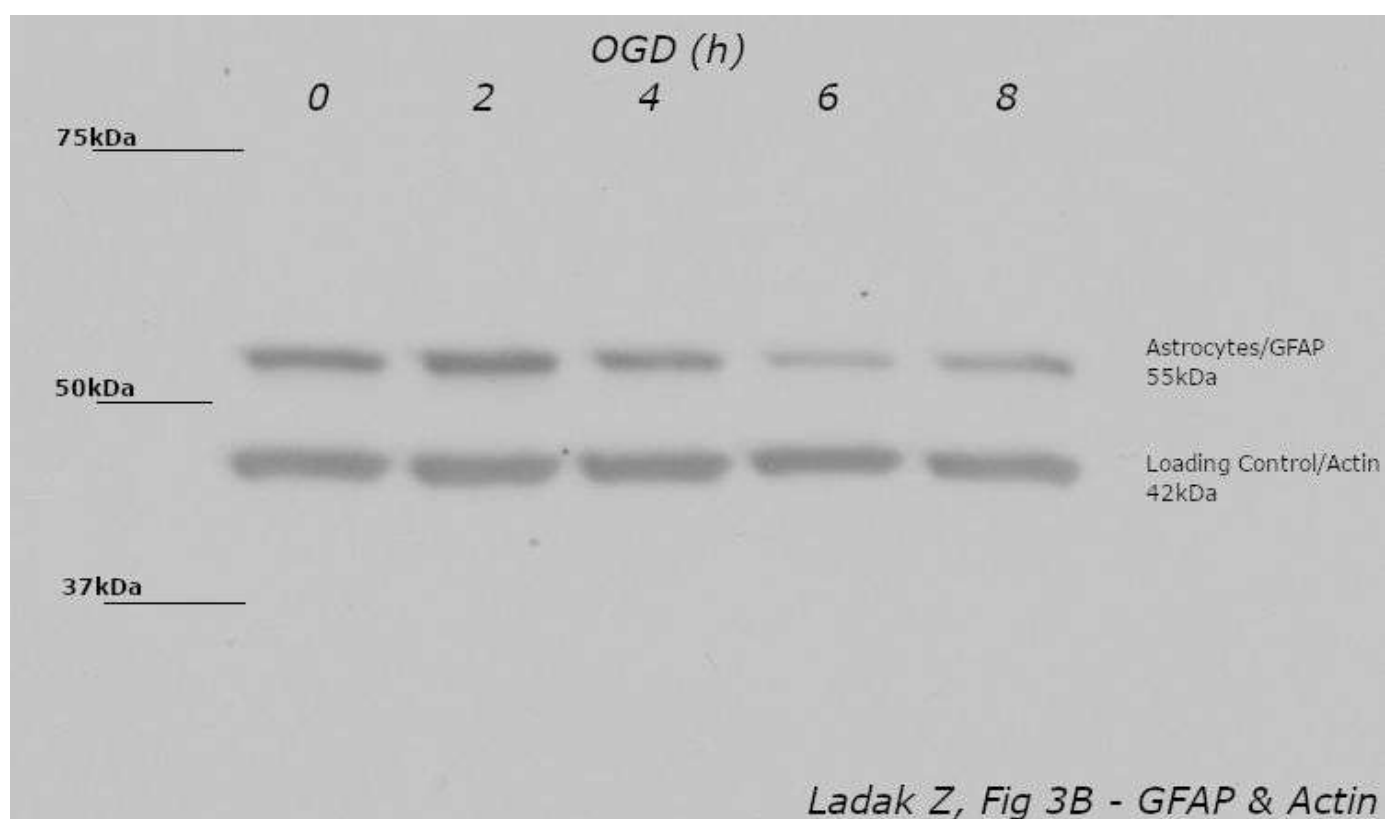

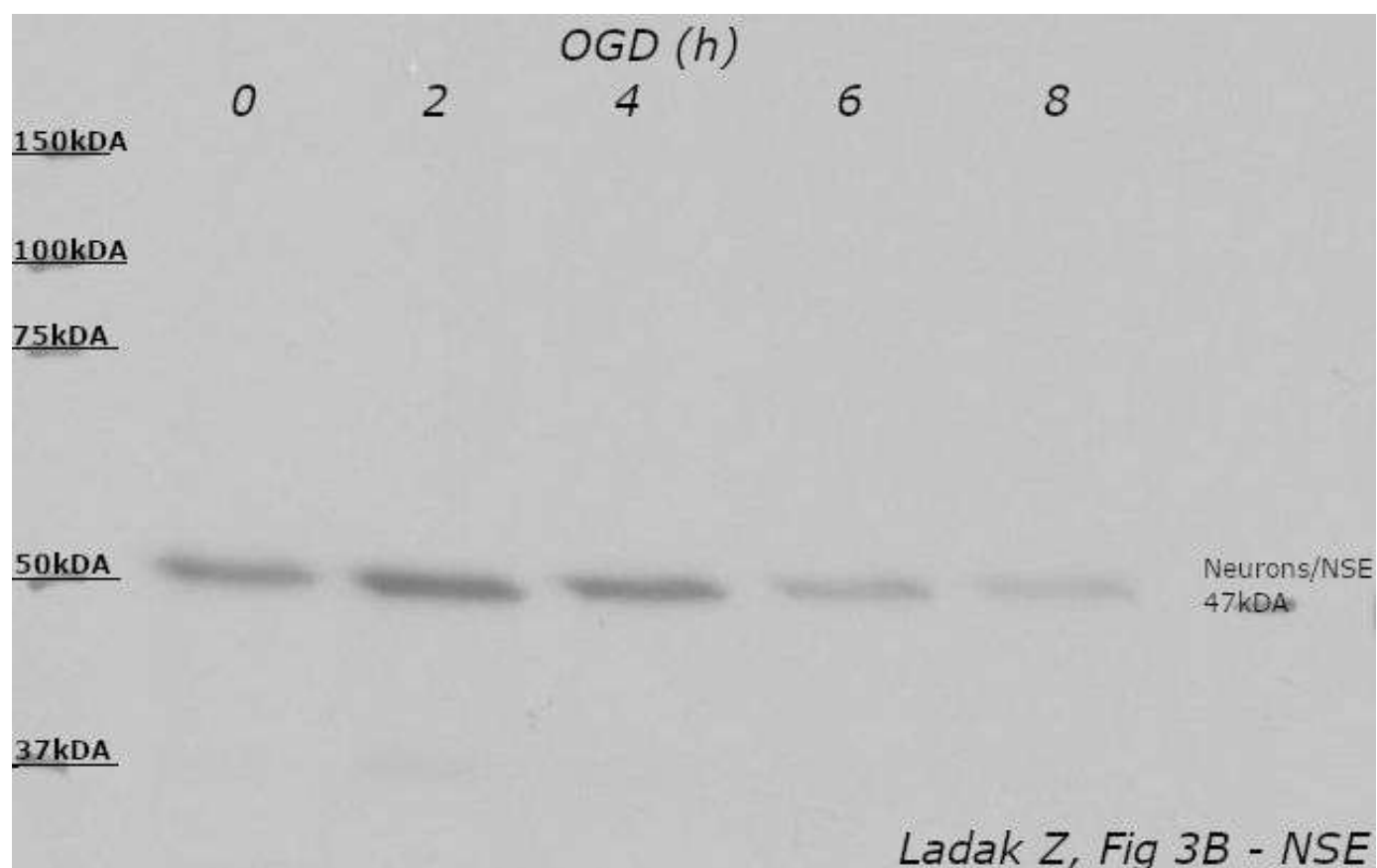

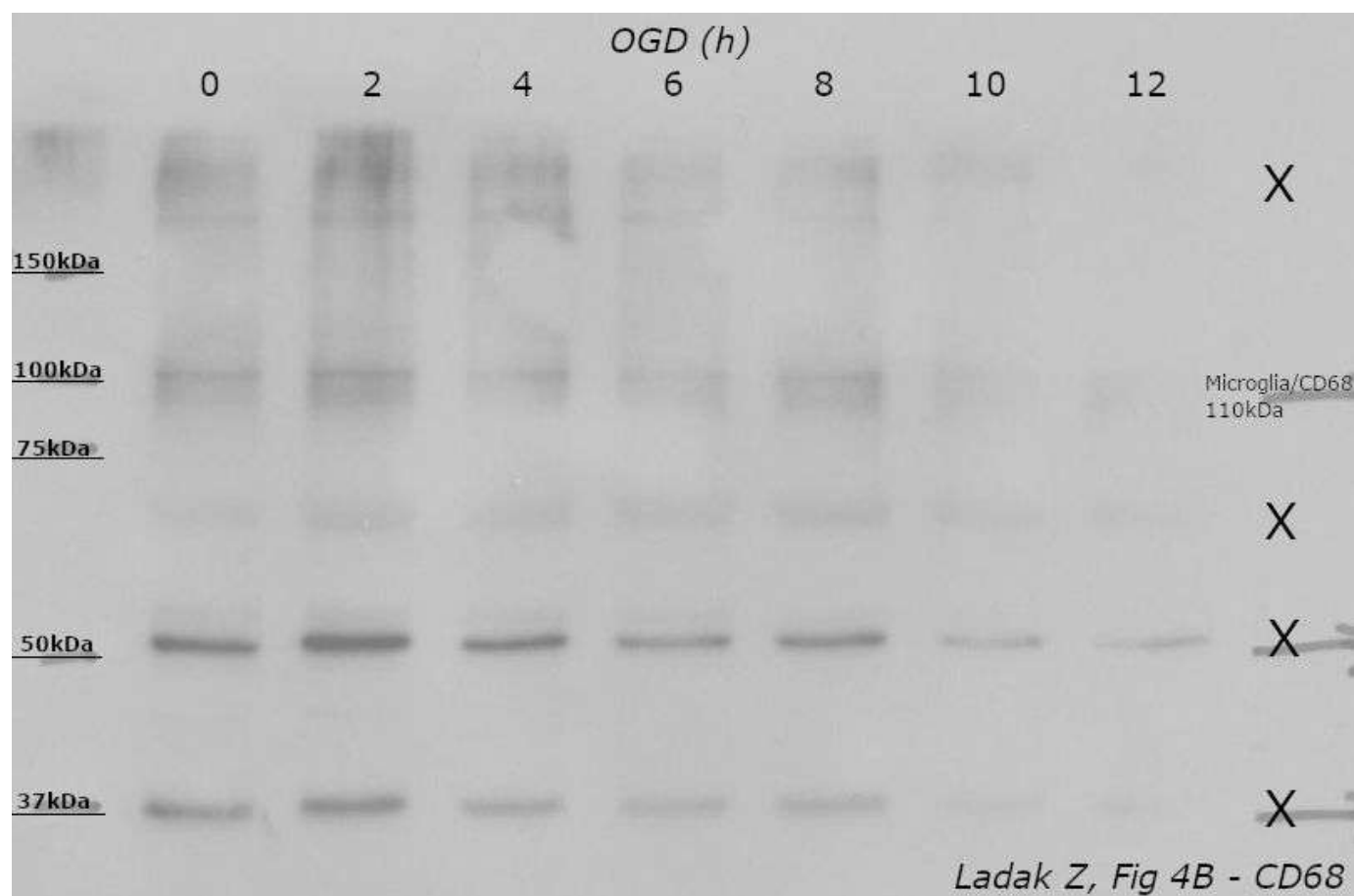

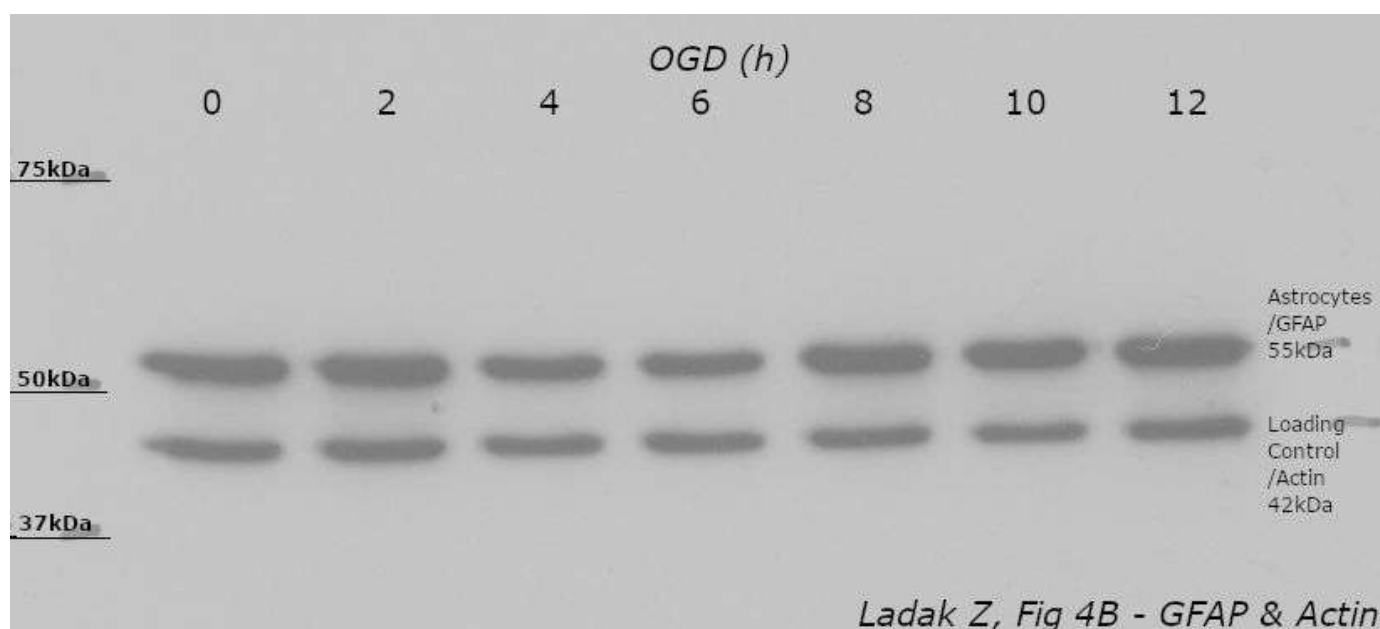

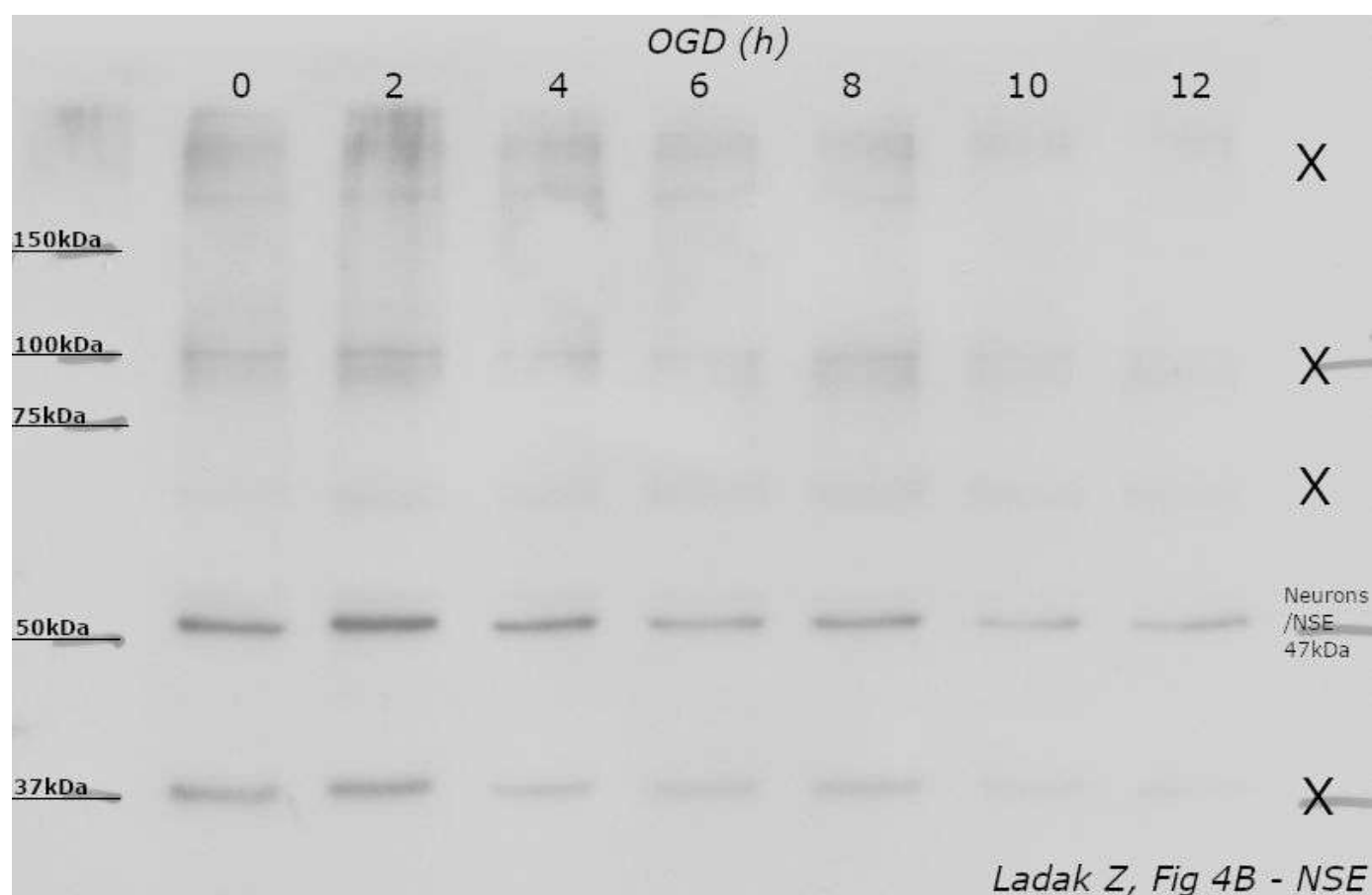

Supplement: S1 Raw images — (PDF) [file pone.0248777.s002.pdf]
